# 3D reconstruction of human movement in a single projection by dynamic marker scaling

**DOI:** 10.1371/journal.pone.0186443

**Published:** 2017-10-18

**Authors:** Erez James Cohen, Riccardo Bravi, Diego Minciacchi

**Affiliations:** Department of Experimental and Clinical Medicine, Physiological Sciences Section, University of Florence, Florence, Italy; University of Chicago, UNITED STATES

## Abstract

The three dimensional (3D) reconstruction of movement from videos is widely utilized as a method for spatial analysis of movement. Several approaches exist for a 3D reconstruction of movement using 2D video projection, most of them require the use of at least two cameras as well as the application of relatively complex algorithms. While a few approaches also exist for 3D reconstruction of movement with a single camera, they are not widely implemented due to tedious and complicated methods of calibration. Here we propose a simple method that allows for a 3D reconstruction of movement by using a single projection and three calibration markers. Such approach is made possible by tracking the change in diameter of a moving spherical marker within a 2D projection. In order to test our model, we compared kinematic results obtained with this model to those with the commonly used approach of two cameras and Direct Linear Transformation (DLT). Our results show that such approach appears to be in line with the DLT method for 3D reconstruction and kinematic analysis. The simplicity of this method may render it approachable for both clinical use as well as in uncontrolled environments.

## Introduction

The clinical assessment of biomechanical parameters is fundamental for both rehabilitation and prevention. As such, the need for objectivity when evaluating appears to be of great importance. However, more than often the assessment of such parameters is still more subjective than warranted, limited to a simple observation by the examiner of the movements performed and scoring the performance according to various clinical scales (e.g., [1–4]). To overcome this difficulty, various instruments exist that allow for more objective measurements. These instruments vary in complexity and in costs, from simple manual goniometers to refined automatic kinematic assessments tools (e.g., [5–6]). However, when evaluating complex multi-segmental movements frequently the use of the more expensive and refined tools is called for.

One of the corner stones of biomechanical evaluation is the dynamic study of the body in its entirety during movement along with a three dimensional (3D) reconstruction, often achieved by means of some acquisition system, from simple video cameras to complex capture systems (e.g., [7–8]). Such evaluation generally requires dedicated spaces and, frequently, trained personnel for its operation. Therefore, the introduction of low-cost, flexible, and simple tools for dynamic analysis and 3D reconstruction of full body movement may provide the basis for a much wider implementation of these types of biomechanical assessments, in both clinical use as well as in uncontrolled environments.

The use of video for kinematic analysis of human movement represents a simple tool for biomechanics studies. While not as accurate as optical capture systems, it does provide easily obtainable valid data at a lower cost and does not require highly trained personnel for its operation (see [9]); therefore, it may satisfy some of the prerequisites for a widespread implementation. One of the issues regarding video analysis is the reconstruction of the movement in a three dimensional (3D) space, commonly requiring the use of at least two cameras. However, while two cameras are able to localize markers in 3D, more than often some of the markers may be hidden during capturing and, therefore, provide partial information and/or necessitate the addition of more cameras, causing an increase in costs. Also, in order to use two cameras, adequate space must be dedicated that allows for a complete acquisition.

Several approaches have been proposed for 3D reconstruction from video cameras. Widely used is Direct Linear Transformation (DLT) that, with a minimum of 6 calibrated markers, is able to link the information provided by two cameras to reconstruct a 3D space [10] (the comparison between the DLT method and other approaches is beyond the scope of this paper; for comparisons and considerations between calibration methods see [11]). This approach, however, does have its downsides. The first already mentioned, is the obligated use of at least 2 cameras. The second is that, when relating image points to object points, a series of constants must be used. These constants for each camera are represented by the projection coordinates, global coordinates, and a series of coefficients that relate the two. Therefore, for each point we have 5 knowns (i.e., 2 for the projection and 3 for global coordinates) and 11 unknowns (i.e., coefficients) per camera. These coefficients, or DLT parameters, are expressed by two equations per projection point. To find these unknown parameters, at least 11 equations are needed, per camera. This can be done by adding calibration points. For each additional calibration point, two new equations are introduced, while the DLT parameters remain the same. Therefore, by using 6 calibration points, which yield 12 equations, we are able to solve for the DLT parameters (for a detailed explanation of the DLT method see [10]). Therefore, to calibrate the system according to the DLT method, a minimum of 6 accurately placed calibration points are needed, and we have to create the transformation matrix for each point, which may result in quite a tedious procedure. Also, when a marker is not visible on one camera the reconstruction cannot be made for said marker.

An appealing alternative is the reconstruction of movement by using a single camera. Not only for the reduction in number of cameras, and therefore costs, but also in cases in which a single camera is used for 2D analysis, a 3D reconstruction may provide additional information from the same recording. An example for this is gait analysis, where only a sagittal view is considered (e.g., [12–13]) leaving the information obtained as partial. A few studies have addressed the issue of 3D reconstruction by a single projection, providing different methods (e.g., [14–17]). Worth noting is the work of Yang and Yuan [18], in which by adding kinematic constraints associated with a human biomechanical model the authors were able to reconstruct a 3D movement from a single camera quite precisely. However, this approach is based on the same principals as the DLT method and therefore requires the solution for 11 parameters. The reduction of the DLT principals to a single camera with the added kinematic constraints as well as the need for anthropometric data further increased the complexity of this method, rendering it less approachable for personnel with no mathematical background. Also, the use of kinematic constraints renders each calibration subject-specific, and not setup specific, which may cause a great increase in the time for preparation and analysis.

Another general issue that merits attention is the use of the commercially available cameras for which, with the advances in video technology, the accuracy of video-obtained data has increased and more low cost alternatives to specialized cameras have emerged. In fact, the use of webcams and action cameras have been successfully implemented for biomechanical analysis of movement [19–20]. For these types of cameras, more than often information relative to the intrinsic properties of the camera (e.g., focal length, sensor specifications) is not readily available and, consequently, some reconstruction methods may not be employed (as also mentioned by [18]).

In a clinical setting, the implementation of an objective biomechanical assessment, is still far from widespread. This may be due to a series of factors. As mentioned earlier, most of the elaborated systems for biomechanical analysis require a dedicated space (e.g., [21]), which is far greater than that found in a typical examination room, let alone at patient’s bedside or during house calls. Even for a two camera setup, the space required to assure visibility of the entire body, though variable between cameras, exceeds that of a common examination room.

In single camera-based approaches space is not an issue. However, the increase in complexity for implementation of these method, due to the reduction in cameras, may greatly limit their usage. When considering that healthcare professionals are concentrated on specific field of expertise, it is not surprising that the most may not possess adequate knowledge or preparation for the application of said methods.

Another general consideration is that the majority of calibration processes are setup-specific, meaning that once the cameras are calibrated they cannot be moved which reduces the mobility of the system and, therefore, may obstacle a common day-to-day use in dynamic environments, such as those found in clinical practice.

In addition, as costs and resources are also to be considered, acquisition of specialized cameras specific for movement analysis is not always possible. Especially today, where most portable devices are able to provide fairly decent video recordings at hand’s reach [22], acquisition of specialized equipment may and should be avoided when possible.

When taking all of these considerations together, it is obvious that in order to render an objective biomechanical assessment widespread, a simple, mobile instrument that is camera independent and does not necessitate any specific background is needed.

Here we propose a simple approach that requires a minimum of 3 markers for calibration and is able to reconstruct movement in a 3D space with a single projection. Such algorithm is based on the scaling effect provided by a two dimensional projection. Seeing that the scaling effect occurs throughout the movement, we called this method dynamic marker scaling (DMS). This approach is independent from the intrinsic properties of the camera and may be widely implemented. In order to test the validity of the DMS method, we compared it to the commonly used DLT method with two cameras.

## Materials and methods

### Subject and task

A normal subject (female, age 25, height 167 cm, 47 kg) was analyzed for linear and angular kinematics of the entire body during a simple lifting task of a box (dimensions 10 x 4 x 2 cm, 100g). No indication was provided to the subject regarding how the task is to be performed. The experimental protocol conformed to the requirements of the Federal Policy for the Protection of Human Subjects (U.S. Office of Science and Technology Policy) and Declaration of Helsinki, and has been approved by the Research Ethics Board of our Institution (Local Ethics Committee, Azienda Ospedaliero Universitaria Careggi, Florence, Italy). The participant provided informed consent in written form.

### Cameras

**Fig 1:**
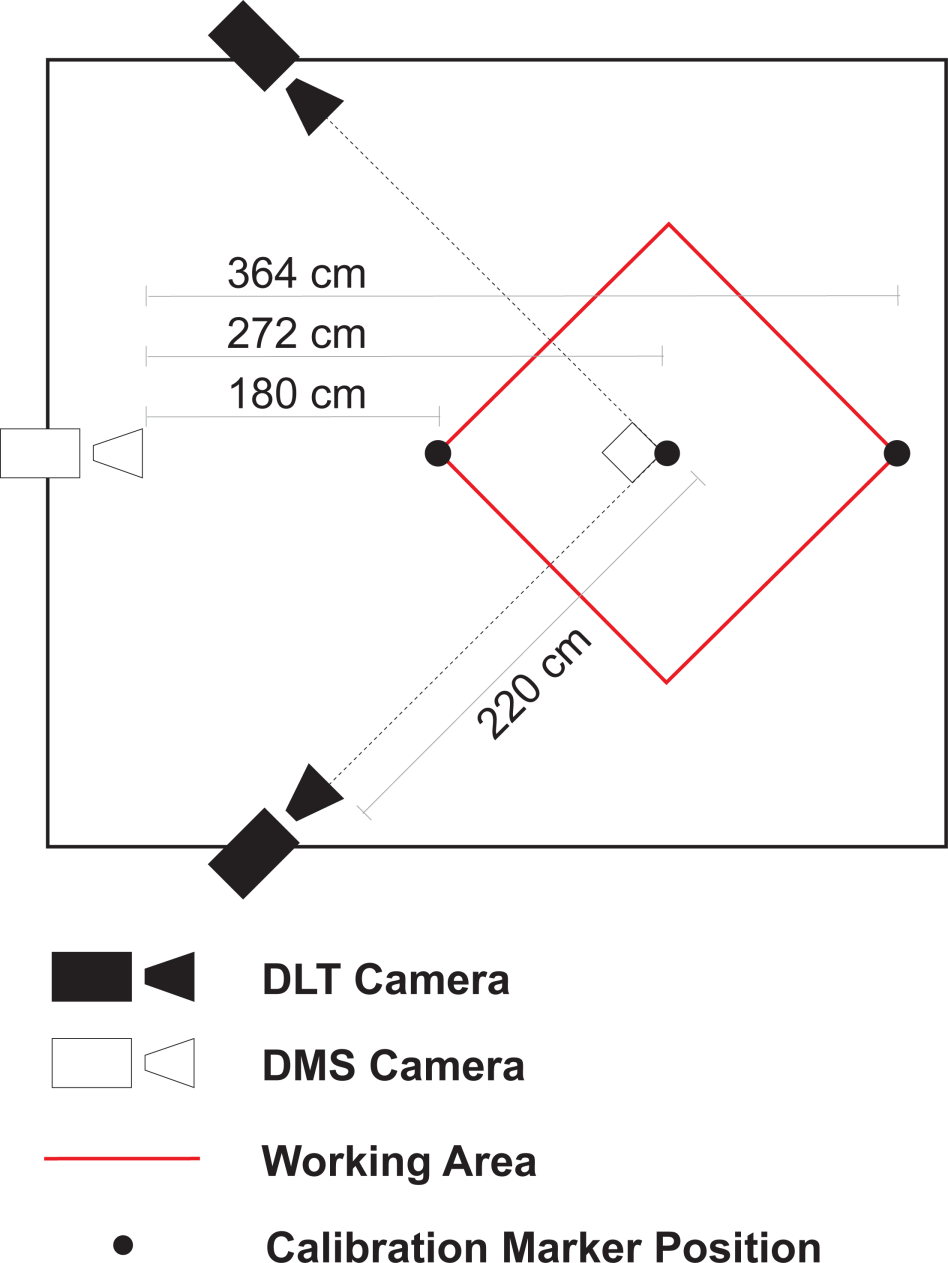
Setup. A diagram of camera placement for acquisition. The cameras used for DLT were placed at a 45° angle from the center point, while the DMS camera was placed frontal to the working area (in red). Also, placement of the calibration markers for the DMS method are shown.

Three cameras were used for data acquisition (GoPro Hero 5 Black), 2 for the DLT method and one for the DMS method. The DLT cameras were positioned orthogonally from one another forming a 45° from the center of the working area at a distance of 220 cm from the center point. The camera used for the DMS method was placed at a distance of 272 cm and frontal to the center of the working area (Fig 1). In order to compare the same movement for DLT and DMS, all three cameras were synchronized by using GoPro Smart Remote control. Video acquisition was set for resolution of 1080p at 120 frames per second for the DLT and DMS cameras. Other settings included: Field of view-Narrow, Color-flat, WB-3000K, ISO-1200, EV Compensation- -0.5.

### Calibration

**Fig 2:**
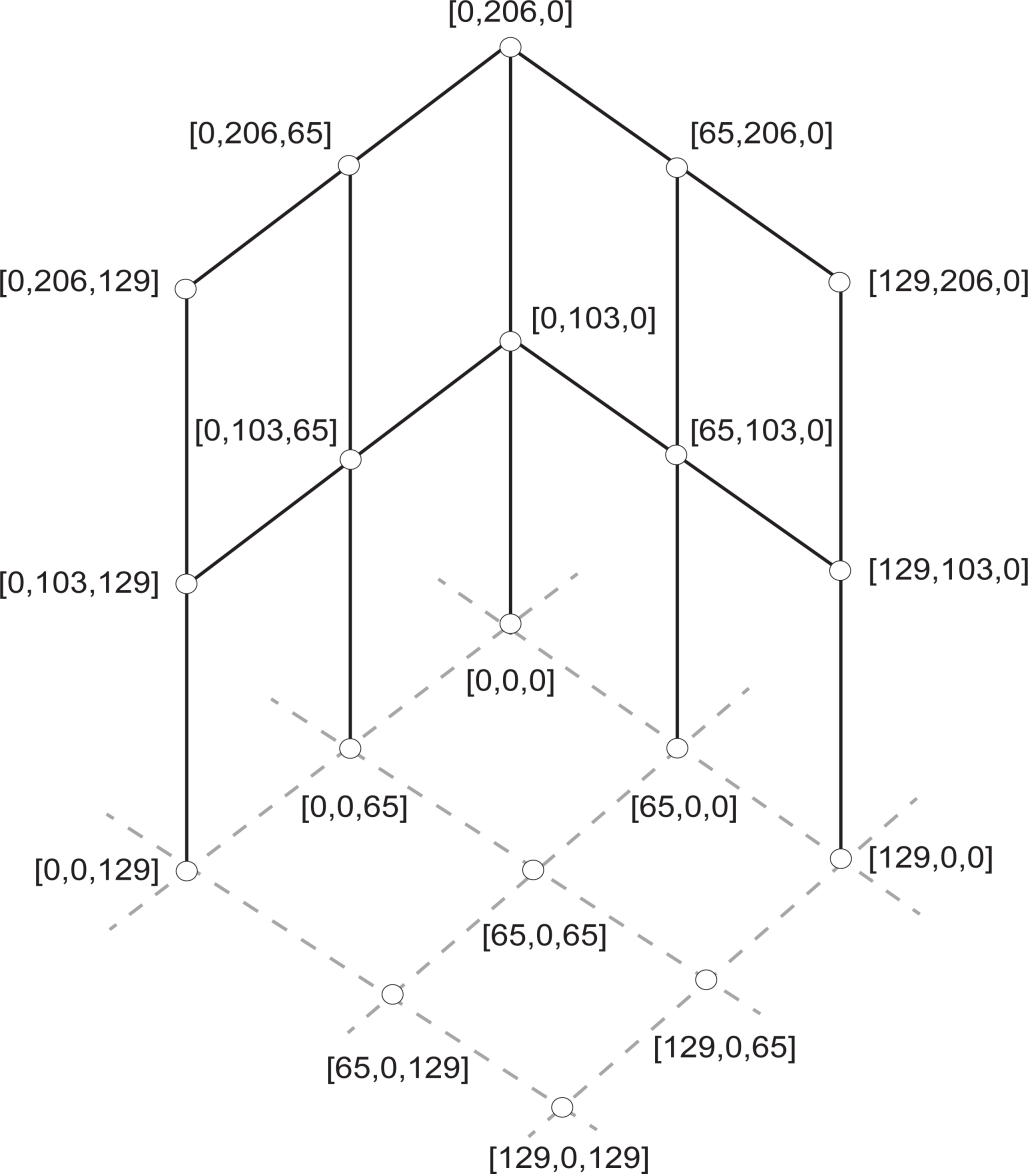
DLT setup. A diagram of the placement of calibration markers for the DLT method. A static structure formed by two fixed orthogonal frames was built to delineate the working area, to which markers were fixed at known positions. For each marker, global coordinates in cm are shown in the parenthesis as XYZ.

**Fig 3:**
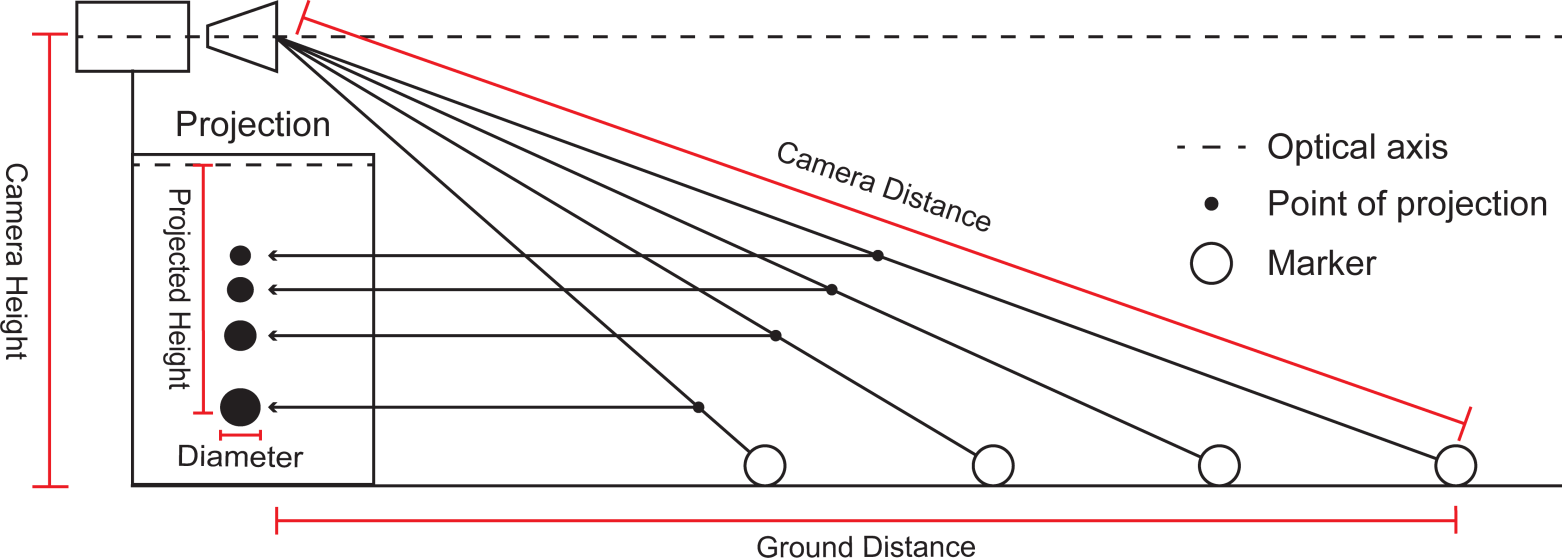
Relationship between position and projections. A diagram describing differences between actual positions of markers, relative to the camera, and their projections. Camera Height is considered as the measured height from the ground to the lens center. Camera Distance is considered as the distance between the marker’s center and the center of the camera’s lens. Ground Distance is considered as the distance from the marker’s center to the camera’s plane. Also shown are differences in projection height, where more distant markers are projected higher than closer ones, as well as diameter changes relative to distance, with closer markers appearing bigger than more distant ones.

**Fig 4:**
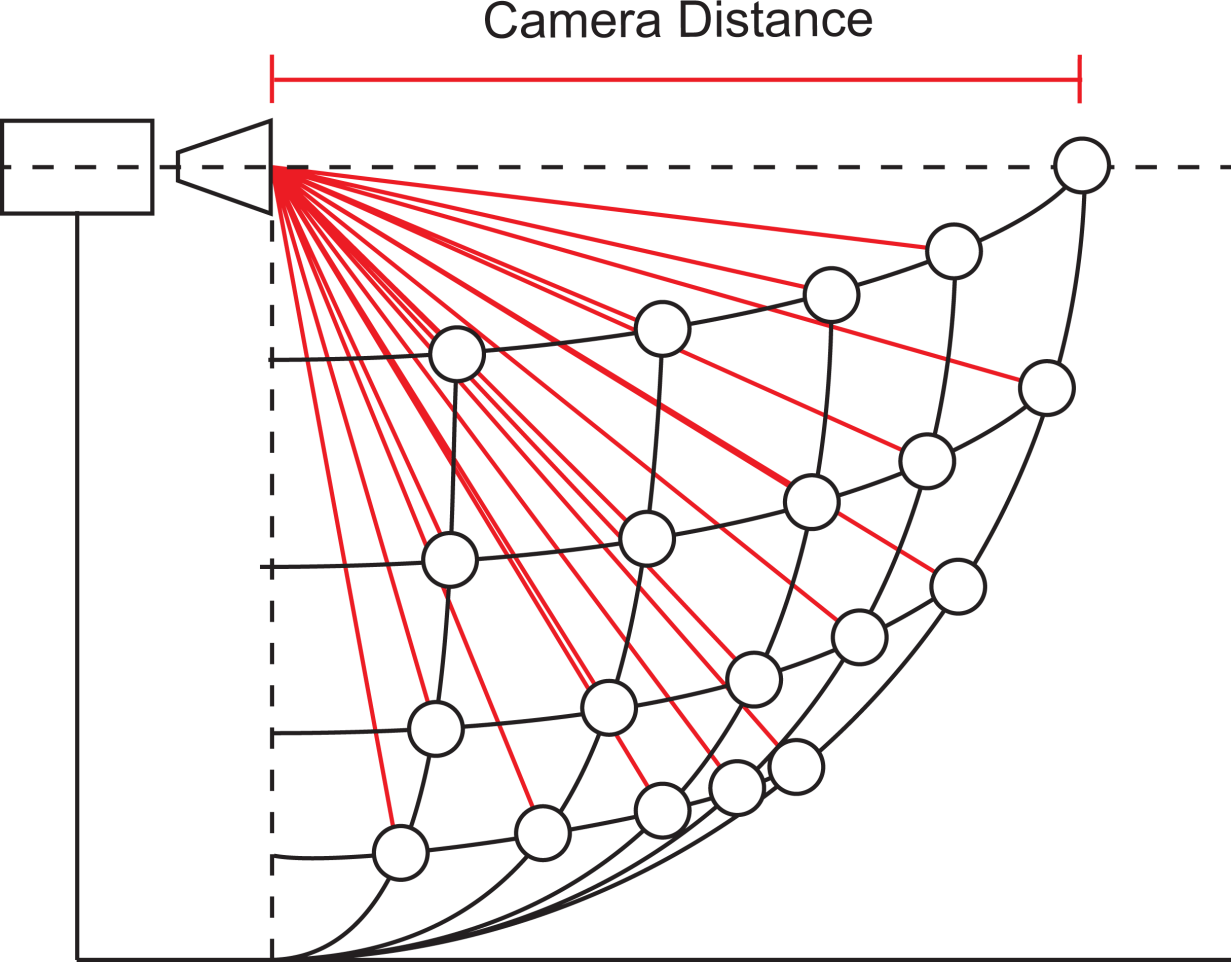
Camera distance. A diagram demonstrating that when a marker measures a certain diameter, said marker will have a specific camera distance independent from its direction. Corresponding camera distances are shown in red lines, all of which are equal to the measured camera distance. An example from our measurements is a projected diameter of 24 pixels, and a corresponding camera distance of 203.56 cm, in any direction.

For DLT calibration 19 spherical markers were placed at known locations, fixed to a static structure thus distributing the markers throughout the working area (Fig 2). For the DMS method, three spherical markers were placed on the ground at known distances from the camera (180 cm, 272 cm, and 364 cm, Fig 1). The distances were chosen to delineate the working area. All of the markers used in this study were 2.4 cm in diameter. For implementation, our algorithm requires the following parameters: marker height (simplified by placing markers on the ground), marker diameter, camera height (considered from ground to lens center, measured at 90.6 cm), marker distance from camera’s plane (i.e., ground distance). Seeing that scaling of objects occur in reference to the center of the camera’s lens, the actual camera distance was calculated from the measured ground distances (as the ground distance and camera height are known, see Fig 3). According to this calculation, a ground marker placed at 180 cm has a camera distance equivalent of 201.51 cm, considering a camera height of 90.6 cm. Therefore, if a marker at 180 cm (ground distance) is known to have a certain diameter when projected, any marker that measures the same diameter can be considered to be placed at a distance of 201.51, in any direction, from the camera’s center (i.e., camera distance; Fig 4).

**Fig 5:**
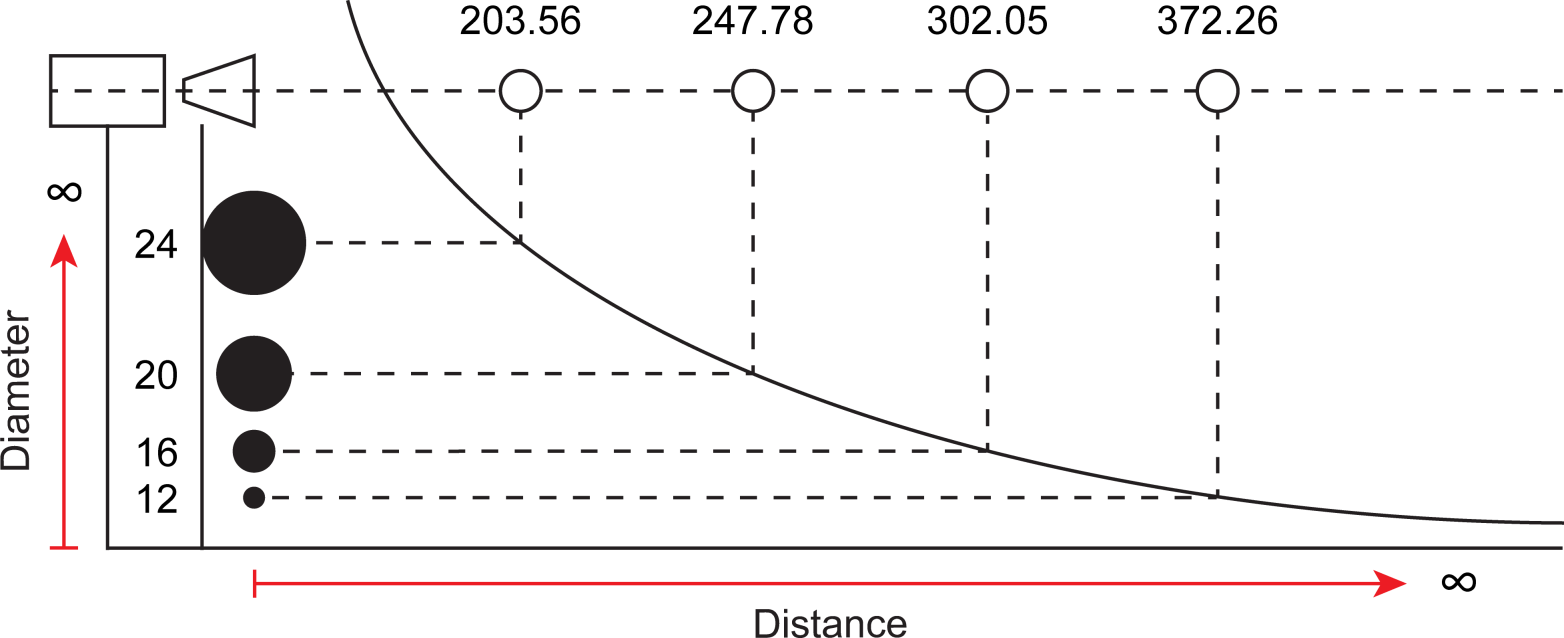
Relationship between diameter and distance. A diagram describing the relationship between a marker’s camera distance and its diameter. The curve of this relationship takes the form of an inverse power function. From this function it is possible to see that the closer the marker is to the camera center, its size approaches infinity and as its diameter approaches zero, the distance grows to infinity. In the figure, we have included also some of the measurements obtained by us relative to the marker’s camera distance (in cm), and its projected diameter (in pixel).

**Fig 6:**
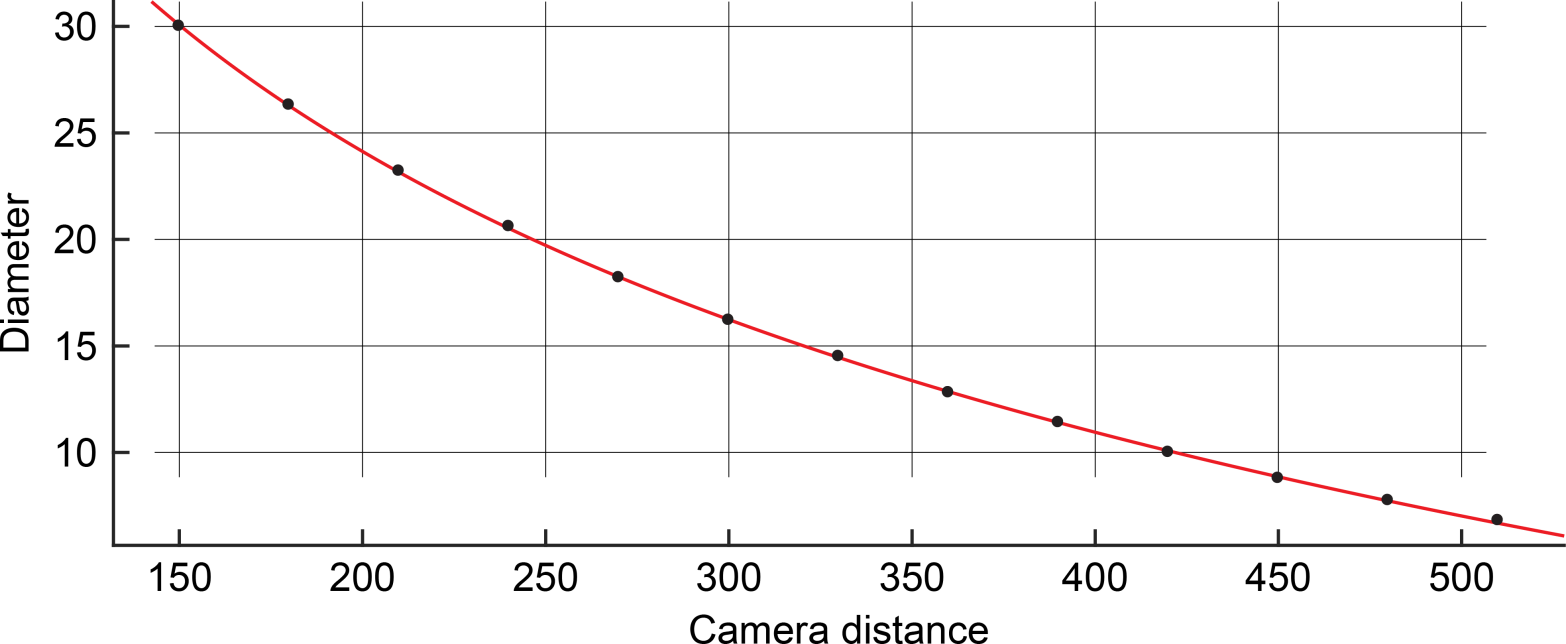
Goodness of fit of function. Plotted values of paired measurements of camera distances (in cm) and corresponding diameters (in pixels). The red curve represents the function that was fitted to the measurements. For the goodness of fit values, see text.

Calibration for conventional cameras is usually based on a perspective projection model, known as the pinhole camera model. While calculation based on a pinhole camera model can be solved for with simple projection equation (e.g., *u = fX/Z*, *v = fY/Z*; where *u* and *v* are the projected coordinates, *X*, *Y*, and *Z* are the real world coordinates, and *f* is the focal length), due to camera parameters that don't match the pinhole model (e.g., large aperture, lens distortion, etc.) as well as our scope to create a camera independent model (i.e., that does not rely on prior knowledge of the intrinsic parameters of the camera, specifically the focal length) the equation needs to be made more general. As such, for a dynamic analysis through marker scaling the following premise was considered. There is an inverse relationship between marker size and its distance from the camera (i.e., marker diameters grows as the distances reduces; Fig 5). Therefore, two asymptotes are present according to these conditions allowing for an implementation of a negative power function based on:

$$f\left(x\right)=x^{-1}$$

where *f(x)* represent the camera distance, and *x* represents the marker’s projected diameter. By including a scaling factor and noise effect the resulting function is:

$$f\left(x\right)=ax^{b}+c$$

where b is a negative number. During the formulation of our model, we have conducted several trials in which markers were placed at different distances, and their projected diameters measured. The results obtained with this function appeared to be in line with our measurements. To test the goodness of fit of our function we used the curve fit tool of Matlab, plotting 13 measurements of distances and diameters. Our calculated coefficients matched those obtained with the curve fit tool, with an R^2^ and adjusted R^2^ values of 0.999, a root mean squared error value of 0.07, and a sum of squared errors of prediction value of 0.04 (see Fig 6).

In order to solve for our equation, seeing that there are 3 unknown coefficients, a minimum number of 3 known points is needed. While the choice to use a power function may be reasonable enough, it is still arguable how accurate this function may be. However, considering the fact that we are interested only in results occurring within a limited numerical range (i.e., working area), and that our calibration markers were placed at the limits and center of said range, measurements obtained by the function are expected to be relatively accurate.

**Fig 7:**
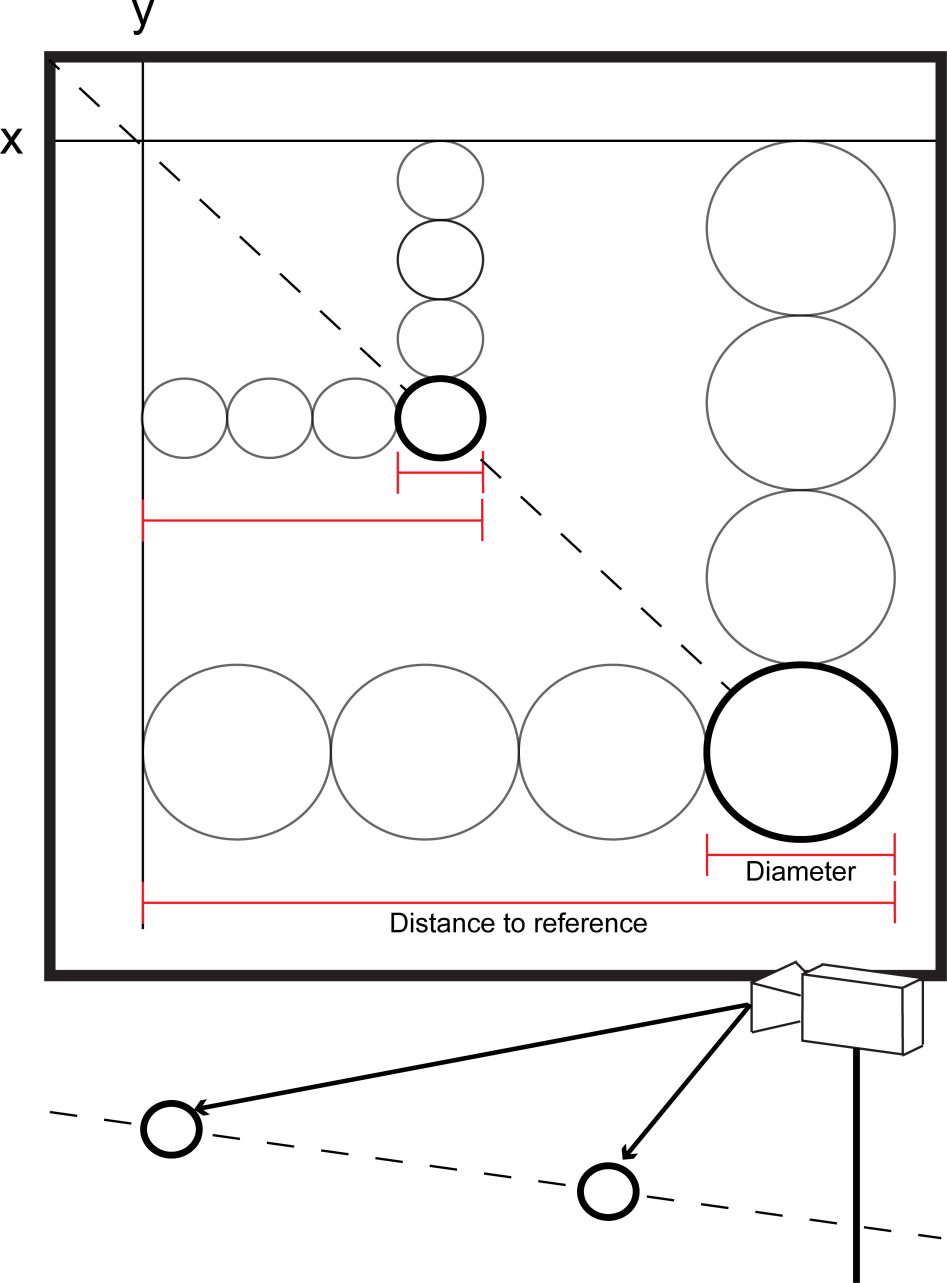
Perspective correction. A diagram describing the correction for perspective distortion. By using the measured projected diameter of a marker as well as the distance to some reference point, it is possible to calculate the ratio between projected diameter and distance to the reference. As the real marker diameter is known, it is possible to multiply the ratio by the real diameter, therefore obtaining the actual distance from the reference. This may be applied to both the X and Y axes. In the diagram we can see two markers (bold circles), that are placed at different distances from the camera (as shown at the bottom of the figure) and that in their projection (bold circles in the frame) appear to have a different diameter (with more distant marker having a smaller projected diameter), and a different localization (with the more distant marker appearing higher and more medial). As we can see the markers are effectively placed one behind the other (in the bottom of the figure) and, in fact, when calculating the ratio between projected diameter and distance to the reference, both present the same ratio meaning that in reality are placed one behind the other (i.e., having the same X and Y global coordinates).

After resolving for the camera distance, a correction factor should be used for the Y and X axes seeing that, due to perspective distortion, objects more distant from the camera appear closer to the center (i.e., optical axis). For example, on the Y axis, more distant objects appear higher when placed below the optical axis of the camera, and lower than they above (Fig 3). On the X axis, more distant objects appear more medial whereas closer objects appear more lateral. In order to resolve for perspective, the known marker diameter can be used. By taking the projected diameter of a marker and calculating the projected distance of that marker from a reference point (for simplicity we used the axes origins), we can quantify that same distance in terms of projected diameter instead of pixels. Seeing that the actual diameter of the marker is known, the conversion of that measurement into centimeters is made by a simple multiplication which could be expressed by the following equation: actual(Y) = (projected(y) /projected(diameter)) * actual(diameter). By using a spherical marker the same approach can be used for both the X and Y axes, this concept is exemplified in Fig 7.

After obtaining data relative to the X and Y axes, a conversion of the measured camera distances obtained into distances from the camera’s plane (i.e., Z axis) is necessary. This conversion can be made by using the Pythagorean theorem, with the measured camera distance and the obtained Y value.

### Data acquisition

**Fig 8:**
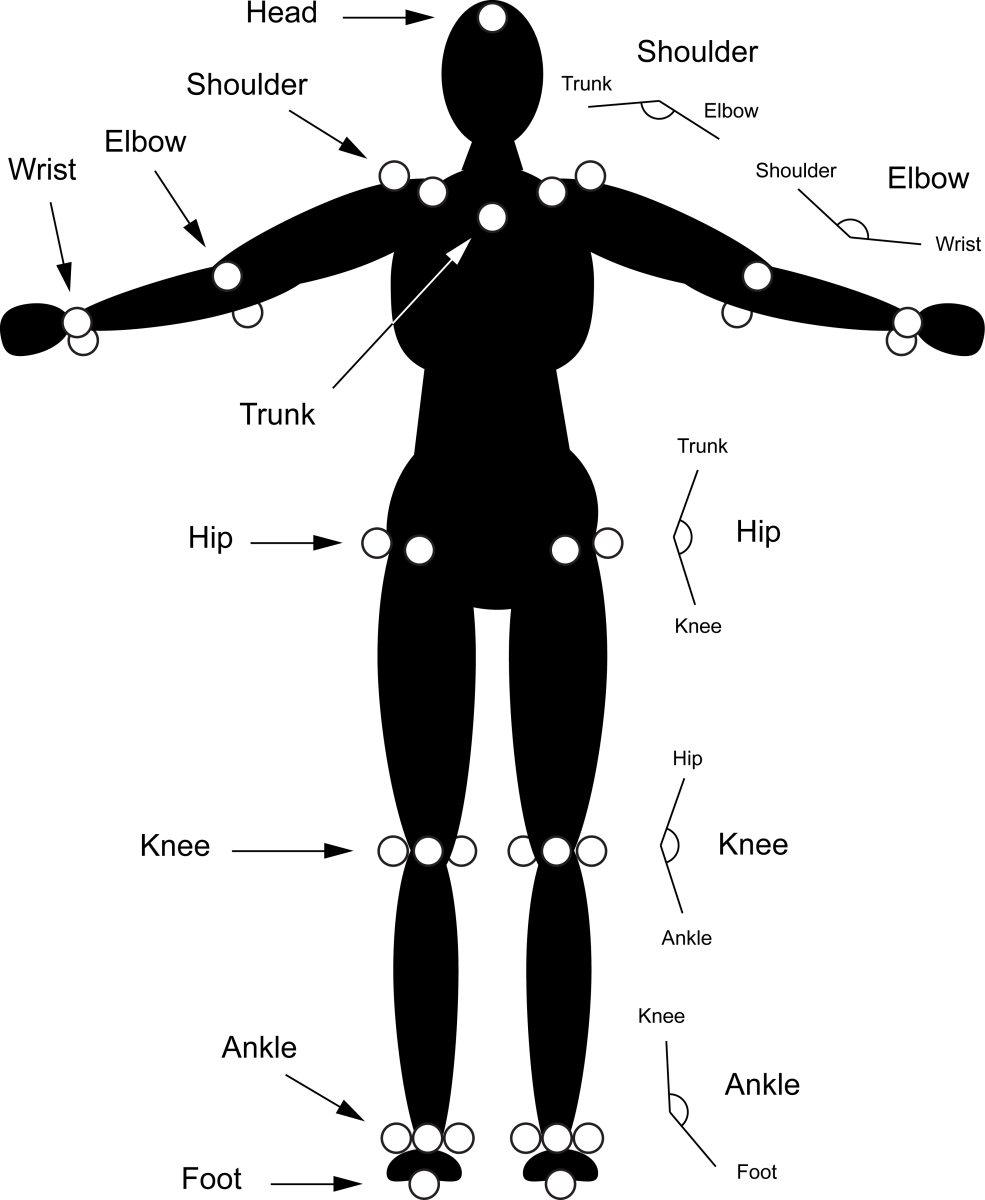
Marker placement. A diagram illustrating marker placement on the subject. As shown, 16 points were taken into consideration with 2 markers per joint for shoulders, elbows, wrists, and hips 3 markers per joint for the knees, and ankles, and a single marker for the head, trunk and left and right feet. Also shown are the joint angles, illustrated in the right part of the diagram; angles are named according to the joint at the vertex.

For a full body analysis of the movement, 12 joints were considered (ankle, knee, hip, shoulder, elbow, and wrist joints) and additional markers were placed on the head, feet and trunk for a total of 16 points of interest (see Fig 8). To assure joint tracking, 2 markers were placed per joint (3 for the knees and ankles). For both DLT and DMS methods, marker tracking was done manually using the open source software Tracker (http://physlets.org/tracker/). The data was extracted from Tracker as x-y coordinates for each tracked point which were then analyzed using Matlab.

**Fig 9:**
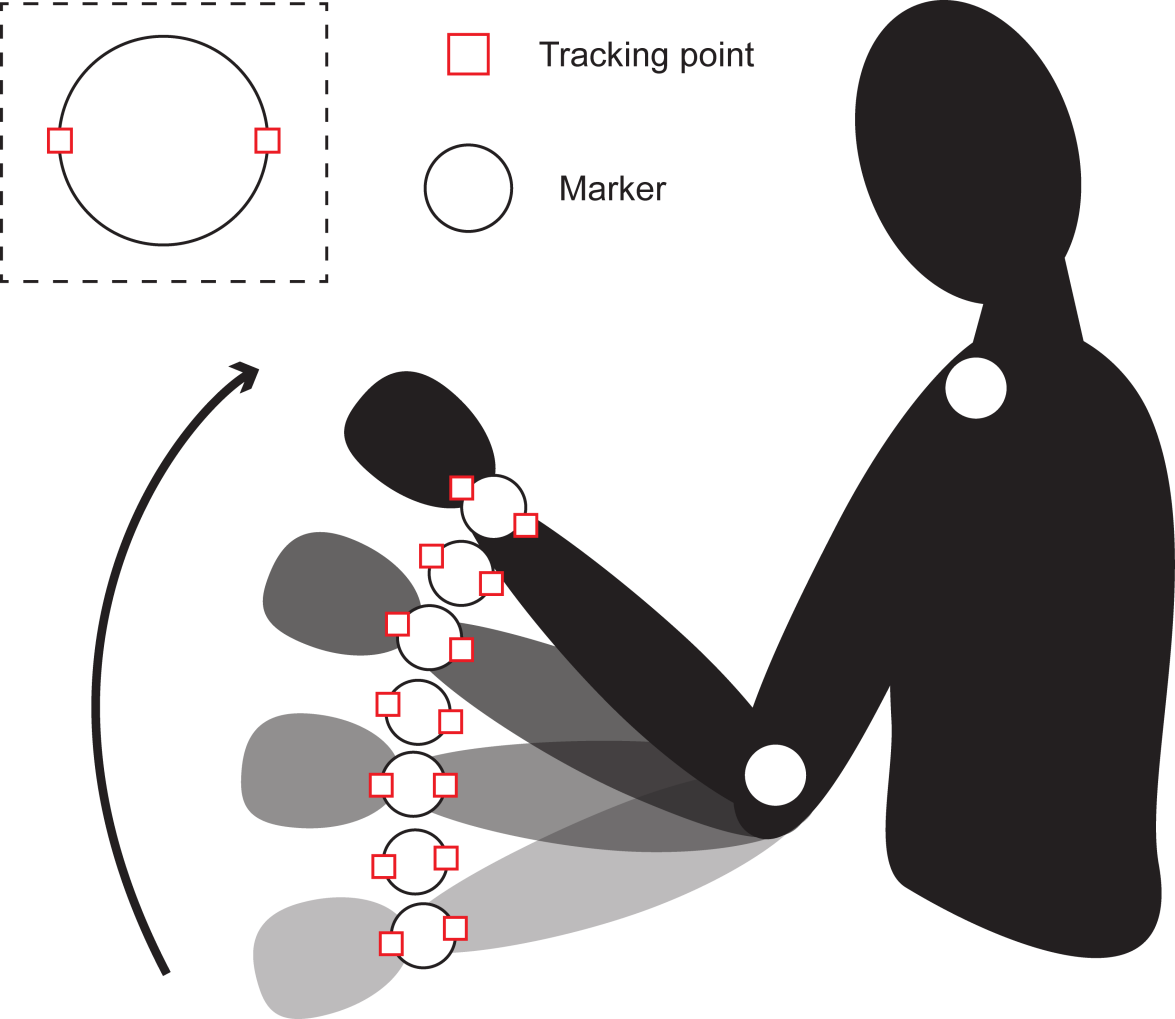
Marker diameter acquisition. A diagram demonstrating marker diameter acquisition. Each side of the marker is tracked for every frame.

For DMS analysis, other than the coordinates of the marker, we are also interested in its diameter and, therefore, tracking points were placed at each side of the marker. Thus, by calculating the difference between the two tracked points, the marker’s diameter may be retrieved (Fig 9). The use of spherical markers for diameter acquisition provides the advantage that the dimensions of the marker do not change with movement. As long as the marker is half visible, data regarding its distance may be retrieved. Also, by using spherical markers, the perspective correction for both X and Y axes is simplified significantly.

### Analysis

The bases of biomechanical analysis from video recorded data is the extraction of kinematic parameters. Of these parameters fundamental data are represented by the change in joints positions and angles over time, from which it is possible to calculate other kinematic parameters such as linear and angular velocities, accelerations as well as a more in depth analysis via inverse dynamics for kinetic data. In order to calculate for the various kinematic parameters, we have constructed an algorithm with Matlab. Seeing that the DLT and DMS methods are calibrated differently, we limited our analysis to linear displacement and angles, both of which are independent from the coordinate system used.

Linear displacement was calculated as the change in position from the starting position for every point in time. In order to resolve for displacement in a three dimensional space, a vectorial calculation is necessary. Therefore, the change in position for every frame was calculated for each axis separately (e.g., x_i_-x_0_). Then the three dimensional displacement for a frame was calculated as the square root of the sum of the changes in position of each axis squared.

Angle calculation was made by taking the 3D coordinates of three points at a time, considering the middle point as the vertex. First, the rays were calculated as the vectors between the first to middle and middle to third points. Then the norm of dot and cross products of the vectors was obtained, and the four-quadrant inverse tangent of the norm was found giving the angle for the three points in radians, which was then converted to degrees.

The following angles were considered for each side of the body: ankle (foot-ankle-knee), knee (ankle-knee-hip), hip (knee-hip-trunk), shoulder (trunk-shoulder-elbow), elbow (shoulder-elbow-wrist), Fig 8.

### Statistics

The coefficient of determination (R^2^) was used to determine the closeness of fit between measurements obtained with the DLT and the DMS methods. The residual differences between the two methods were measured for each joint in order to quantify the magnitude of the differences, reported here as mean, standard deviation (SD), and maximal residual difference (RD). Although usually used to determine the noninferiority or equivalence between treatments [23], we believe that an equivalence test may help to better characterize the level of similarity between the two methods. Therefore, a Two One-Sided Test (TOST) was implemented to better define the equivalence between the two methods [24]. Seeing, however, that the equivalence margins are not defined in current literature for this type of analysis, we have used the equivalence test to find said margins. This way, we hope to provide at least some quantification of the accuracy between measurement, which may benefit future studies in this field.

## Results

**Fig 10:**
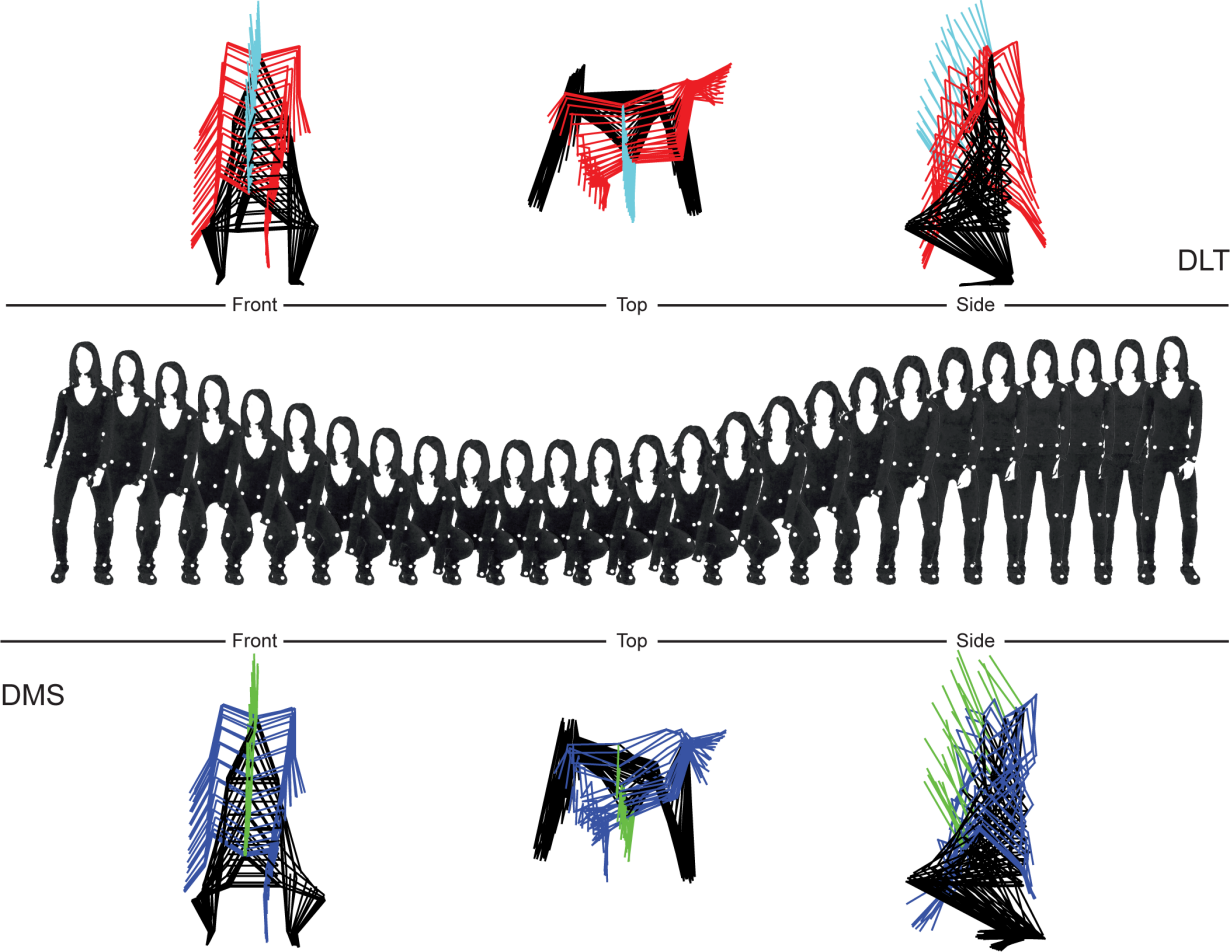
A reconstruction of the movement from both the DLT and DMS methods. The actual action, as sequenced images is displayed along with the reconstruction for each method. For simplicity, only 1 every 10 frames is shown for reconstructions and image sequence. Reconstructions are shown in three different points of view: front, top, and side views. To differentiate between body segments, different colors were used for the lower extremities (black), upper extremities (red for DLT and blue for DMS) and head (cyan for DLT and green for DMS).

**Fig 11:**
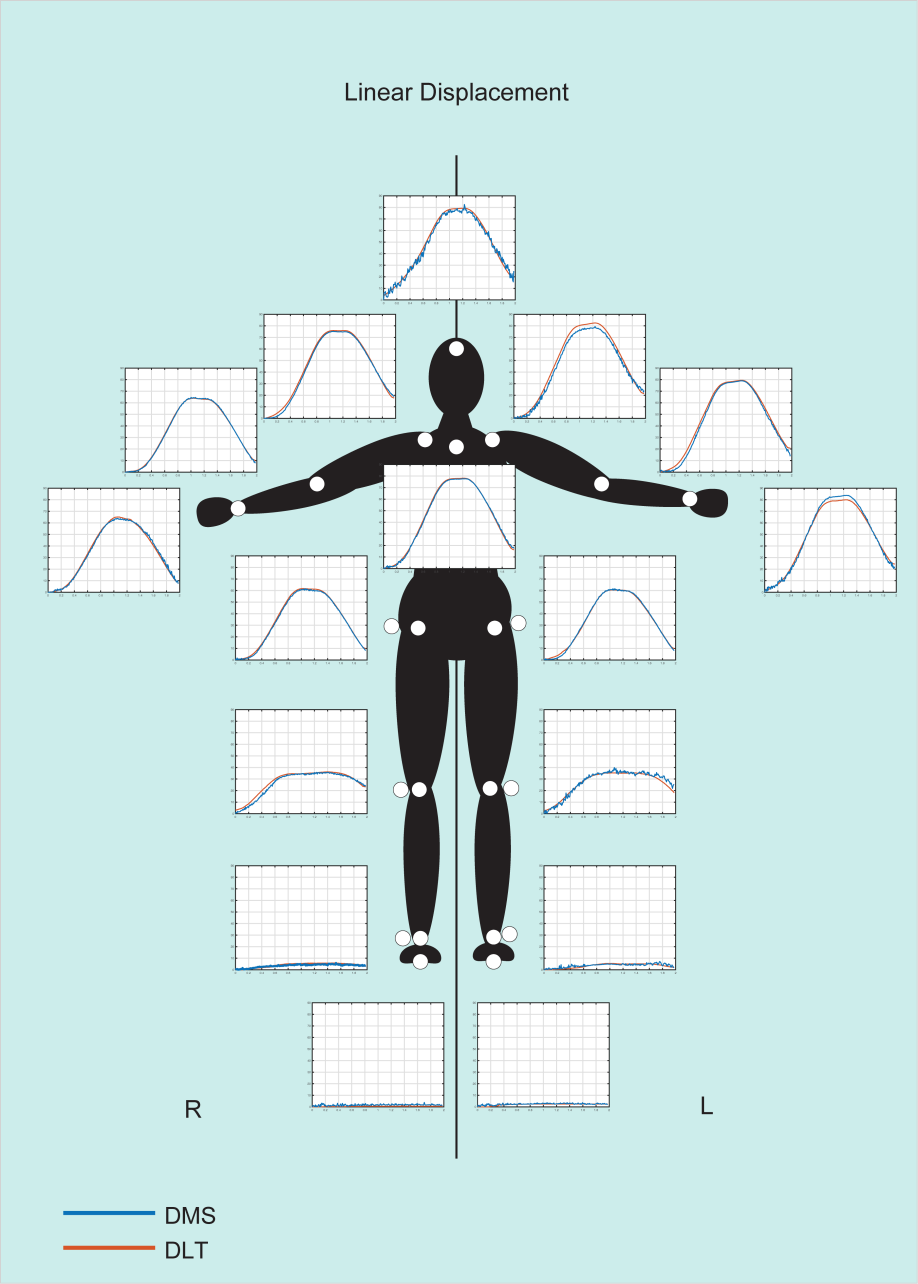
Comparison between the DLT and DMS methods for linear displacement. DLT results (red lines) and the DMS results (blue lines) are shown in the graphs. Graphs represent the amount of displacement for each joint (in cm, from 0 to 90 cm) over time (in seconds).

**Fig 12:**
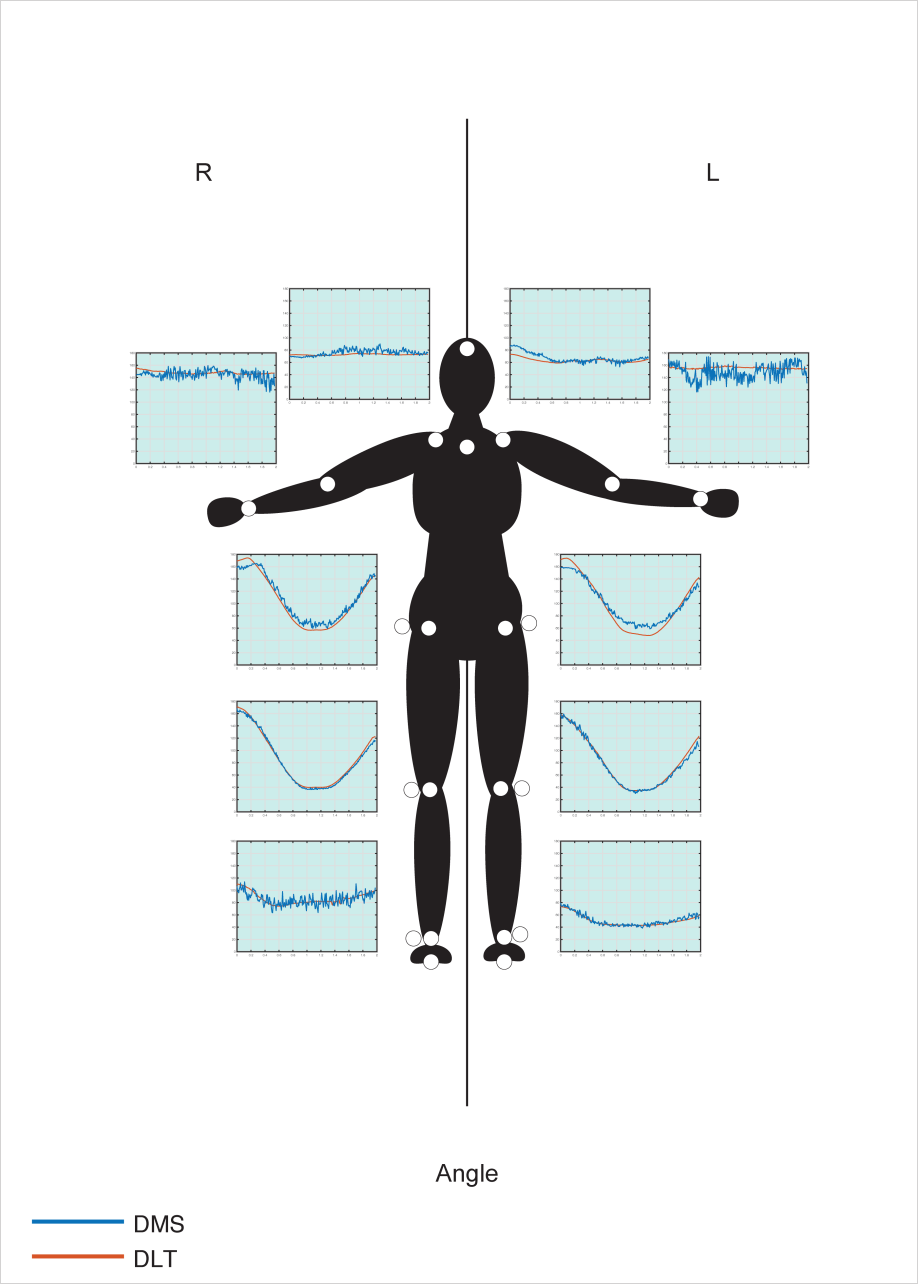
Comparison between the DLT and DMS methods for angles. DLT results (red lines) and the DMS results (blue lines) are shown in the graphs. Graphs represent the angle measured (in degrees, from 0 to 180) over time.

The two approaches, overall, provided relatively similar results (for raw data see S1 Data). The 3D reconstructions acquired from both methods along with the image sequence of the real movement are shown in Fig 10 whereas graphical representations of the results are shown in Fig 11 and Fig 12.

For linear displacement, R^**2**^ values were all above 0.9 (ranging from 0.92 to 0.99), with the exception of the left ankle (measuring 0.7), right and left foot (measuring 0.14 and 0.53 respectively). Mean RDs ranged from 0.45 to 3.3 cm with SDs ranging from 0.35 to 1.53 cm (see Table 1). Also, TOST values were found to be significant for all points when the equivalence margins were set to ±7.2 cm. The margins were found after repeated measurments revealed equivalence between methods for all points, considering a p-value of 0.05.

**Table 1:** Results linear displacement.

	R^2^	Mean RD	Max RD	SD
**Head**	0.990813957	2.307647273	7.677104063	1.395137606
**Trunk**	0.998847845	0.810182236	2.505493549	0.545043738
**Left Shoulder**	0.996118777	3.305988917	5.989332726	1.539170798
**Left Elbow**	0.997861284	2.101304731	6.974407234	1.383657008
**Left Wrist**	0.997102118	2.000816104	4.204164705	1.236915977
**Left Hip**	0.998946363	0.654426565	2.743673593	0.581703567
**Left Knee**	0.967712171	1.578760936	7.21099502	1.530080016
**Left Ankle**	0.702763426	0.840581915	3.879585996	0.693594929
**Left Foot**	0.537222438	0.497938173	2.719627706	0.500363313
**Right Shoulder**	0.997738755	1.49221282	3.544471991	0.830858065
**Right Elbow**	0.999648089	0.452151143	2.335514054	0.321224971
**Right Wrist**	0.996187449	1.149458162	4.028278309	0.793175871
**Right Hip**	0.999374197	0.830437528	2.015402446	0.530943298
**Right Knee**	0.98790825	1.520426534	4.902626691	1.223279492
**Right Ankle**	0.92790915	0.477757197	1.492372869	0.350301012
**Right Foot**	0.143197359	1.211692724	3.292572228	0.570023829

For angles, R^2^ values were more dispersed (see Table 2), with highest values measured for the hips, knees, and left ankle (ranging from 0.91 to 0.99). Intermediate values were measured for the two shoulders and right ankle (ranging from 0.22 to 0.73), whereas low values were measured for left and right elbows (0.01 and 0.025, respectively). Mean RDs were all under 10 degrees with SDs values measuring less than 6 degrees, with the exception of the left elbow which measured a mean RD of 11.64 (SD 8.32) degrees. TOST values were found to be significant for all angles when the equivalence margins were set to ±10.009 degrees after repeated measurements revealed equivalence between methods for all angles, considering a p-value of 0.05.

**Table 2:** Results angles.

	R^2^	Mean RD	Max RD	SD
**Left Shoulder**	0.73507208	4.870967733	15.86678296	4.470245747
**Left Elbow**	0.002792385	11.64482273	38.03087703	8.327434338
**Left Hip**	0.984469344	9.486651945	19.84794478	5.228241367
**Left Knee**	0.98927716	3.080016315	16.07554307	3.17898316
**Left Ankle**	0.919177284	2.396324697	10.58431677	1.818507089
**Right Shoulder**	0.255002155	4.490444506	16.24981011	3.373191805
**Right Elbow**	0.011336711	6.714043036	30.18082833	5.089619732
**Right Hip**	0.977301432	7.298045535	18.70282298	4.203696301
**Right Knee**	0.994615467	3.100617972	11.07574791	1.970521106
**Right Ankle**	0.466757649	6.158030253	23.52001801	4.709636293

## Discussion

For the most part, the results obtained with the DMS method appear to be in line with the DLT method. For linear displacement, greater differences were present for less mobile joints (i.e., feet and ankles). However, when considering the mean RDs, they were all under 1.5 cm. This difference is relatively low, especially when considering that the highest mean RD was registered for the left shoulder (measured 3.3 cm). This is further emphasized when considering the SDs, which were all low for both feet and ankles (0.35–0.69 cm) along with maximal RD of 1.49–3.87 cm. Compared to other joints, highest SD was registered to the left shoulder (1.54 cm), and highest maximal RD measured to the head (7.76 cm). This type of trend is to be expected seeing that measurements for less mobile points are more susceptible to small differences, especially when considering that both methods are an approximation of the real values, and as such, are more likely to present greater differences for fixed points.

For angles measured between the two methods, the data also seem to suggest that for the less active joints (in angular terms), results differ greatly compared to the more active joints. In fact, according to their R^2^ values, the joints may be divided into three groups in terms of activity: highly active (knees, hips, and ankles), moderately active (shoulders), lowly active (elbows). The exception in this case was the right ankle, which presented an R^2^ value of 0.46, however, when looking at the graph it is evident that this value is mostly due to dispersion (Fig 12). The level of activity of the joints may also be seen by the sequenced images of the movement, in which the elbows appear to be at a relatively stationary angle compared to the other joints, followed by the shoulders (Fig 10). When examining the graphs obtained from the angular measurements, it is possible to see that the general trend appears to be very similar between methods (Fig 12), with greater data dispersion for the DMS method compared to the DLT method. As for linear displacement, also in this case it appears that as the measurement in question is more stationary, greater differences ensue. With that in mind, still all of the mean RDs between methods were under 10 degrees, with the exception of the left elbow measuring at 11.64 degrees. Such difference may easily be attributed to relatively smaller differences between the position of joints, where even slight movements may greatly influence the angles measured, which is further magnified the more stationary the measurement is.

Some general limitations provided by both the DLT and the DSM method should be noted. As pointed earlier, video analysis produces less accurate results compared to other systems, such as optical capture systems [9]. Also, it is well known that a marker-based measurement may result in inaccuracies due to inaccurate placement, skin movement, attachment on loose clothing etc. In fact, alternative markerless-based approaches are emerging to overcome these difficulties [25].

As for specific limitation of the DMS method, as demonstrated by the graphs, is that measurements for the DMS method present a greater dispersion of data. This is mostly due to the fact that a more precise measurement is needed in order to retrieve the markers diameters and, by being a pixel-based measurement, it is more likely that the diameters measured will be skewed from one frame to another, especially when objects are more distant or less mobile. Moreover, dispersion of data may be the result of inaccuracies in acquisition due to contrast issues within the video, which may limit the visibility of contours of the markers. The importance of adequate contrast between marker and surrounding is emphasized also in other works (e.g., [25–26]). In fact, in our experience a higher dispersion of data was found for the joints in which contrast between the marker and the surroundings was lowest (i.e., head, trunk, wrists).

These inaccuracies of the DMS methods may, however, be substantially reduced by increasing the resolution of acquisition as well as the frames per seconds. The new commercial cameras, such as action cameras, provide a resolution up to 4K at 30 frames per second (reduced when frames per second are increased), which is sufficiently high to reduce measurement errors. Also, a manual tracking of the markers, instead of the automated algorithms of various software, may further increase the accuracy of the method. Finally, data dispersion may be reduced either by applying adequate filters or data smoothing.

Still, it should be considered that the DLT method also has its own inherent errors as the transformation from R2 to R3 based on only a few markers remains as only an approximation, which may be reduced by increasing the number of calibration markers. Worth mentioning is the fact that in this study we compared the DLT method calibrated according to 19 points to the DMS method, calibrated with only three diameters.

## Conclusions

The DMS method appears to provide relatively similar results compared to the DLT method, at least when gross movements are concerned. This method may be used alongside the DLT method in cases in which markers become hidden in one of the cameras. This way, by calibrating the cameras also according to the DMS method, data relative to said marker may still be salvaged. Also, the algorithm presented here may be of value for acquisition of data in specific tasks such as gait, that when is studied with a single camera, only the sagittal plane is considered (e.g., [12–13]). This way information that may be obtained from a frontal plane is eliminated. Perhaps the biggest advantage of the DMS method is that the entire calibration process is very simple compared to other approaches of 3D reconstruction with a single camera, or multiple cameras in general, which translates in rapidly obtained data. Also, the use of a single camera and three markers renders it much more mobile than other methodologies. This may be of value especially when the goal of the measurments is to provide a general estimation of the movement rather than a precise description. As pointed out in a recent review by Hewett and Bates [27], preventive biomechanical interventions may help in reducing the incidence of various musculoskeletal injuries, referred by the authors as “preventive biomechanics”. Therefore, having some measurement in a clinical setting may help in identifying those people who might benefit the most from preventive interventions. The simplicity and mobility of the DMS method may render it as an adequate instrument for widespread this type of clinical use or in other uncontrolled environments.

## Supporting information

S1 Data Raw data recorded for both DMS and DLT methods. (ZIP)
